# Age differences and brain maturation provide insight into heterogeneous results in autism spectrum disorder

**DOI:** 10.3389/fnhum.2022.957375

**Published:** 2023-02-02

**Authors:** Kirsten O’Hearn, Andrew Lynn

**Affiliations:** ^1^Department of Physiology and Pharmacology, Atrium Health Wake Forest Baptist Medical Center, Winston-Salem, NC, United States; ^2^Department of Special Education, Vanderbilt University, Nashville, TN, United States

**Keywords:** MRI, maturation, adolescent, gray matter, white matter, social, cognition, face

## Abstract

Studies comparing individuals with autism spectrum disorder (ASD) to typically developing (TD) individuals have yielded inconsistent results. These inconsistencies reflect, in part, atypical trajectories of development in children and young adults with ASD compared to TD peers. These different trajectories alter group differences between children with and without ASD as they age. This paper first summarizes the disparate trajectories evident in our studies and, upon further investigation, laboratories using the same recruiting source. These studies indicated that cognition improves into adulthood typically, and is associated with the maturation of striatal, frontal, and temporal lobes, but these age-related improvements did not emerge in the young adults with ASD. This pattern – of improvement into adulthood in the TD group but not in the group with ASD – occurred in both social and non-social tasks. However, the difference between TD and ASD trajectories was most robust on a social task, face recognition. While tempting to ascribe this uneven deficit to the social differences in ASD, it may also reflect the prolonged typical development of social cognitive tasks such as face recognition into adulthood. This paper then reviews the evidence on age-related and developmental changes from other studies on ASD. The broader literature also suggests that individuals with ASD do not exhibit the typical improvements during adolescence on skills important for navigating the transition to adulthood. These skills include execution function, social cognition and communication, and emotional recognition and self-awareness. Relatedly, neuroimaging studies indicate arrested or atypical brain maturation in striatal, frontal, and temporal regions during adolescence in ASD. This review not only highlights the importance of a developmental framework and explicit consideration of age and/or stage when studying ASD, but also the potential importance of adolescence on outcomes in ASD.

## Introduction

Samples with autism spectrum disorder (ASD) display different developmental trajectories when compared to typically developing (TD) samples. These distinct trajectories lead to group differences between ASD and TD samples that change with age, and these age-related changes are evident in behavior, brain function, and brain structure. Thus, collapsing across a large range of ages or controlling age as a nuisance factor when examining ASD can be misleading, leading to inconsistencies in the literature even when groups are well-matched. On the other hand, assuming that the developmental trajectories are likely to be altered in ASD provides insight into developmental mechanisms that work differently in ASD, including the systematically different environments and experiences that people with ASD encounter.

This paper reviews how the interconnected factors of age/developmental stage/cohort (simply referred to as “age” throughout the paper) impact behavior and brain function and structure—and alter research results—in ASD. We first assess age-related changes in our local studies and then report analyses from other studies that also assess age-related or longitudinal changes in those with ASD and TD peers. Differential development between older children with and without ASD may be particularly evident with social cognition and skills. Research on ASD focuses on these skills because impaired social skills are diagnostic of ASD. However, social cognition and skills may also be unique in that, typically, their development continues well into adulthood, and to a high level of expertise which is relatively consistent across TD individuals (Gauthier and Nelson, 2001). The deficits in ASD and this prolonged development may interact to make social development proceed atypically in ASD. Thus, we argue that age should be considered as a factor of interest in studies of ASD, with few exceptions. Of course, this level of detail may not always be possible, as it requires larger samples and adds complexity to analyses. However, with increased data sharing, more studies may be able to examine age directly. In addition to age-related variability, samples with ASD also exhibit increased individual variability in behavior and in brain function, compared to TD samples (Magnuson et al., 2020; Heller Murray et al., 2022). This individual variability undermines our power to detect developmental change, adding to the difficulty in understanding differential development in ASD.

This paper has several important limitations. While puberty is critical for adolescent development, and gender differences are striking in those with ASD, these topics are beyond the scope of this paper (see Corbett et al., 2020; Geier and Geier, 2021; Wood-Downie et al., 2021). Another limitation of this review is that—for simplicity’s sake—we do not include studies that assess how performance on one task predicts performance on another task (Lai et al., 2017; Gardiner and Iarocci, 2018; Fong and Iarocci, 2020), including a study with longitudinal methods (Pugliese et al., 2016). Finally, we focus on individuals with IQs in the typical range or higher in this review, as these samples are most often included in studies of cognition and neurophysiology.

### Adolescence and adulthood in autism spectrum disorder (ASD)

#### Risks and stressors during adolescence

The subjective “adolescent experience” is likely to systematically differ for children with ASD. Adolescence is an unique stage of development, with distinct social demands and mental health concerns (Pfeifer and Blakemore, 2012; Galván, 2017), and these issues are likely to be amplified in individuals with ASD. Having an ASD appears to increase a number of adolescent risks and stressors that an individual experiences, undermining development into adulthood (Carter et al., 1998; Kanne et al., 2011; Bal et al., 2015). Adolescents with ASD are at a higher risk for problems with social health (fewer, less intimate friends; Orsmond et al., 2013; Magiati et al., 2014) and mental health (anxiety, depression, schizophrenia, substance use; Taurines et al., 2012; Lever and Geurts, 2016; Park et al., 2018; Hossain et al., 2020; Schwartzman and Corbett, 2020; Oakley et al., 2021; Sun et al., 2021), compared to TD adolescents. These heightened risks are often specific to adolescence, and may make adolescence particularly difficult to navigate for those with ASD. Adolescents with ASD are also more likely than TD adolescents to experience stressors, such as bullying (Maïano et al., 2016), social isolation (Orsmond and Kuo, 2011; Orsmond et al., 2013), and health-related risky behaviors (Sun et al., 2021), including suicide attempts (Zahid and Upthegrove, 2017; Kirby et al., 2019; Hirvikoski et al., 2020). These stressors disrupt mental health and brain maturation in TD samples (Page and Coutellier, 2018; Shaw et al., 2020). Thus, a better understanding of this transition—from school age children to young adults—in those with ASD is critical to our goal of transforming this transition from a time of increasing problems to a time that sets the stage for improved adult outcomes (Gillberg and Schaumann, 1989; Hendricks and Wehman, 2009; Newman et al., 2011; Shogren and Plotner, 2012; Wei et al., 2013, 2015; Anderson et al., 2018).

#### Adolescent development in ASD

Adolescence is a time when many individuals become independent. However, the transition to independence can be difficult for those with ASD, because daily living skills (i.e., adaptive behaviors) are particularly problematic in ASD. Daily living skills may improve from childhood to adolescence in ASD (Bal et al., 2015), but seem to plateau during adolescence and early adulthood in ASD (Clarke et al., 2021; Auld et al., 2022; but see Smith et al., 2012 for later plateau in adulthood). Daily living skills and executive function (EF) in ASD are worse than expected on the basis of IQ, and the gap between these skills and IQ increases from adolescence into adulthood (Rosenthal et al., 2013; Pugliese et al., 2015; Kraper et al., 2017; Tillmann et al., 2019). Individuals with ASD appear to use additional resources (e.g., EF skills like attention, planning) for everyday tasks, compared to TD peers, as reflected by significant correlations between EF and daily living skills in a group with ASD but not the TD group (Pugliese et al., 2015). Adults with ASD continue to struggle with independent living throughout the lifespan (Howlin and Moss, 2012; Shattuck et al., 2012; Duncan and Bishop, 2015; Anderson et al., 2021).

#### Adult outcomes in ASD

Adult outcomes tend to be poor in individuals with ASD. Poor physical health outcomes include premature morbidity (Vohra et al., 2017) and mortality (Hirvikoski et al., 2016). Not surprisingly, social and mental health also tend to be negatively impacted by ASD. Adults with ASD are less likely to be employed or to engage in social activities compared to individuals with other developmental disorders (Shattuck et al., 2012; Orsmond et al., 2013). Children and adults with ASD are also at increased risk for mental health problems. In one study, about 72% of adults over 40 with ASD had at least one other psychiatric diagnosis (Bishop-Fitzpatrick and Rubenstein, 2019; see also Lugo-Marín et al., 2019; Hossain et al., 2020). These mental health issues can interact with autistic traits, escalating problems. For instance, ASD may contribute to the etiology of depression, which often emerges during adolescence (Rai et al., 2018; Shephard et al., 2019); in turn, depression undermines social motivation and social cognition, amplifying autistic traits and increasing the social isolation that characterizes ASD (Donohoe et al., 2012; Samamé, 2013). Thus, problems that emerge during the adolescent transition are likely to contribute to poor outcomes throughout the lifespan in ASD (Henninger and Taylor, 2013; Ratto and Mesibov, 2015; Skaletski et al., 2021).

### Social and non-social cognition in ASD

The most widely recognized characteristic of ASD is the deficits in social cognition and resulting difficulties in social interactions (Sasson et al., 2013; Patriquin et al., 2016). Thus, it is often assumed that deficits in ASD will effect social cognition (i.e., memory for, attention to, interpretation of social stimuli) but not “non-social” cognition. However, there are also cognitive differences with non-social tasks and stimuli in ASD. These differences are often characterized as general deficits in executive function (EF), decision making, cognitive control, and/or memory (Hill, 2004; O’Hearn et al., 2008; Rosenthal et al., 2013). Such deficits should impact both social and non-social tasks. Despite extensive study, the overlap across social and non-social cognitive differences in ASD has proven difficult to characterize, often leading to contradictory conclusions (Dakin and Frith, 2005; Behrmann et al., 2006; Parish-Morris et al., 2013; Weigelt et al., 2013). Understanding and comparing the typical and atypical developmental trajectories provides insight into why social cognition is sometimes impaired while non-social cognition is not. Possibilities include: 1. cognitive differences in ASD impact social stimuli more – or differently – than non-social stimuli; 2. social stimuli are more complex or require increased global processing compared to non-social stimuli, and this difference affects those with ASD more than TD individuals; and/or 3. processing of social stimuli improves over a longer period of time (from earlier to later), or to a greater level of expertise across TD individuals, compared to processing of non-social stimuli, and these maturational processes are impacted by ASD. These possibilities are likely to overlap. More detail on age, development and other causes of heterogeneity is needed to clarify which differences in ASD are specific to social tasks, and which generalize to cognitive tasks in general.

## Our studies and studies using the same recruiting source

TD individuals displayed prolonged improvements on face recognition, into adulthood, while individuals with ASD did not. This resulted in group differences between those with ASD and TD peers by adulthood.

### Behavioral studies

Face recognition is the quintessential social cognitive task, and begins developing in the first few hours of life (Walton et al., 1992; Pascalis et al., 1995). We first observed differing trajectories in adolescent development of face recognition on the Cambridge Face Memory Test (CFMT; O’Hearn et al., 2010), originally designed for adults with prosopagnosia (Duchaine and Nakayama, 2006). Unexpectedly, our results indicated that the TD group improved when recognizing faces from adolescence (ages 13–17) to adulthood (18–28), while the sample with ASD did not. This led to group differences between adults with ASD and their TD peers. Similar deficits on the CFMT in adulthood have also been reported in parents of children with ASD (Wilson et al., 2010) and adults with Asperger’s syndrome (Hedley et al., 2011). We later learned that face recognition performance on the CFMT improves until age 30 typically (Germine et al., 2011). This pattern of emerging deficits in adults with ASD compared to TD peers has since been replicated using a modified CFMT in the MRI scanner (Lynn et al., 2018; O’Hearn et al., 2020) and with an eye tracker (Fedor et al., 2018) in our laboratory. Eye tracking results indicated that atypical developmental trajectories on face recognition in ASD do not simply reflect an epiphenomena of eye movement differences, such as eye movements changing with age typically but not in ASD (Fedor et al., 2018).

### Differences between the adult group with ASD and TD adult group were not specific to faces or social stimuli, but instead to tasks that developed into adulthood typically

To understand whether these differences in ASD were specific to faces, or generalized to recognition of individuals of other object kinds, we administered a memory task for faces and cars to a larger sample. We used the immediate memory (IM) task from the Let’s Face It! Battery (Wolf et al., 2008; Tanaka et al., 2010). Using this task, children (8–12) and adolescents (13–17) with ASD exhibited impaired face recognition, but similar car recognition, compared to their TD peers. Developmentally, performance on face recognition improved from childhood to adolescence in *both* the ASD and TD groups (O’Hearn et al., 2014). This pattern differed from the CFMT, possibly reflecting that the IM task was designed for grade school-age children. In contrast, neither group displayed developmental improvement from childhood to adolescence on car recognition, with no differences between individuals with and without ASD or between children and adolescents. Thus, face recognition improved from childhood into adolescence, but car recognition did not, in both groups, making development similar across groups. In contrast, from adolescence to adulthood, development differed between groups. Memory improved for both face and car recognition from adolescence to adulthood in the TD group but neither recognition task improved from adolescence to adulthood in the group with ASD. Thus, deficits were evident by adulthood in ASD on both face and car recognition, compared to TD adults. However, the group difference in adulthood was more robust for faces than it was for cars, potentially reflecting more or longer improvement typically that did not occur during development in ASD (O’Hearn et al., 2014).

One explanation for the late development in car recognition is that memory for individual cars improves typically as adolescents learn to drive, and drive more, into adulthood. This increased car expertise may result in psychological and neurophysiological representations for cars that are more similar to faces than they were previously (i.e., more global, individually recognized, with activation in the Fusiform Gyrus, Gauthier et al., 2000; Gauthier and Nelson, 2001; Ross et al., 2018). This increased expertise may not occur, or may occur later, in adolescents with ASD, as individuals with ASD are less likely to drive, and learn to drive later, compared to TD individuals (Curry et al., 2018).

### Individuals with ASD do not display many of the improvements into adulthood evident in TD individuals

This pattern – of late-emerging differences between groups with and without ASD – also occurred in a dynamic change detection task examining attention. In this task, we hypothesized that the group with ASD would be less sensitive to changes on socially related stimuli, but not changes on non-social stimuli, compared to the TD group. Instead of this expected difference in performance with social and non-social stimuli in ASD, we again found that performance in younger individuals was similar, regardless of whether they were diagnosed with ASD or not. However, performance improved into adulthood in the TD group but not the group with ASD. This led to deficits in ASD in adulthood. The differences between adults with ASD and TD adults were most robust on marginal object changes (compared to conceptually or visually central objects), but there were no group differences between social and non-social stimuli (O’Hearn et al., 2011a). Earlier studies that recruited through the same source (Autism Center of Excellence at the University of Pittsburgh, Pittsburgh, PA) also suggest a lack of adolescent development in ASD. These studies from other laboratories use a variety of cognitive tasks, including recognition of facial expression (Rump et al., 2009), drawing the Rey-Osterrieth figure (Kuschner et al., 2009), and increasing reliance on global (vs. local) processing with age in a letter recognition task (Scherf et al., 2008).

### Individuals with ASD displayed “typical” development from adolescence to adulthood on a WM task

There were a few exceptions to this pattern of results in our laboratory, as described in the previous section. Adults with ASD performed *better* than adults with TD when counting concentric squares (O’Hearn et al., 2013), consistent with the less global, perhaps “immature,” visual processing reported previously in ASD (Happe et al., 2001; Dakin and Frith, 2005; Mottron et al., 2006). This “local processing” strength is associated with better performance in some samples with ASD, compared to TD samples, on tasks that require processing of individual features or other analytical skills (e.g., embedded figures test; Bolte and Poustka, 2004; Dakin and Frith, 2005; Mottron et al., 2006; Ashwin et al., 2009). In addition, on a WM task, TD groups and their peers with ASD also performed similarly, even across age. We tested short-term memory for rapidly presented colors and shapes using a change detection task (Awh et al., 2006). This study did *not* result in group differences at any age between the group with ASD and the TD group. In addition, both groups (ASD and TD) displayed *developmental improvements into adulthood* (Lynn et al., 2022). These strengths and intact skills are important to identify and encourage, as they provide insight into how to better incorporate and support neurodiversity. Neurodiversity contributes unique views and innovative solutions needed to address current problems (Austin and Gary, 2017; Karmakar and Sarkar, 2020; Marciano et al., 2022), and may be a key construct underlying societal growth (Chapman, 2021).

### Neuroimaging studies

#### Neuroimaging results from our laboratory align with the behavioral findings

Neuroimaging studies done in our laboratory also reveal atypical trajectories of brain maturation in those with ASD, compared to TD individuals, and are incorporated into the brain function sections (p. 6 to 7). Briefly, studies of functional connectivity in our sample, one during rest and one during the CFMT task, indicated that brain connectivity *increased* from adolescence to adulthood in the group with ASD, while it *decreased* in the TD group (Padmanabhan et al., 2013; Lynn et al., 2018). This typical pattern is consistent with extant literature suggesting that neurophysiological connections become more specialized during adolescence. In addition, we analyzed brain activation in the temporal lobe, and reported that a lack of developmental change in ASD led to group differences between adults with ASD and TD adults (O’Hearn et al., 2020).

## Literature assessing longitudinal and age-related change

This literature, collected by the first author as it emerged from well-established sources such as the NIH intramural program (Wallace et al., 2010), also provides evidence of atypical adolescent development and brain maturation in adolescents with ASD. Additional searches were done in 2022, and these included the keyword “autism” and one or two of the following words: “adolescence,” “longitudinal,” “development,” “age,” and “meta-analysis.” The focus of this search was on longitudinal studies, meta-analyses and larger samples that directly examined age-related changes on a particular skill or function.

### Behavioral studies

#### Executive function (EF), decision making, and cognitive control

Deficits in EF, decision-making, and cognitive control have long been reported in ASD (Hill, 2004; O’Hearn et al., 2008). However, little is known about how these deficits might change from adolescence to adulthood in ASD. Young children with ASD exhibited deficits in memory and metacognitive skills, compared to TD children. In addition, the impairments in ASD increased with age when compared to TD peers (5–8; Rosenthal et al., 2013). Consistent with this result, a longitudinal study indicated that parent reported EF (i.e., BRIEF; Guy et al., 2004) was not only impaired in those with ASD but also did not improve over 2 years of development, while the TD group exhibited improvements over the same 2 years (initial ages 7–14; Vogan et al., 2018). In contrast, a study examining digit span and standard EF tasks (CANTAB^®^, 2019) reported that the initial deficits in planning and task switching *decreased* from childhood (8–12) to adolescence (13–18) in individuals with ASD, compared to TD individuals, while deficits in memory remained stable across that age range (8–18) (Chen et al., 2016). It is unclear if this pattern would continue into adulthood.

#### Social cognition, communication, and emotional sensitivity

Parent report (i.e., Social Responsiveness Scale-Second Edition) indicates that social communication improves with age in TD individuals from childhood to early adulthood but *declines* with age in individuals with ASD (4–29; Wallace et al., 2017). A longitudinal study also reported that verbal working memory improved over 2 years in TD individuals and individuals with Attention Deficit Hyperactivity Disorder (ADHD) but not in individuals with ASD (9–16; Andersen et al., 2015). Consistent with these studies, a test of social cognition using a theory of mind task in which triangles behaved intentionally (e.g., chasing) indicated that children with ASD fall further behind with age on this task, as TD children improve more with age than those with ASD (2–21; See Figure 3 in Bal et al., 2013). In contrast to this result, a similar theory of mind task indicated that social behavior did not differ between TD individuals and their peers with ASD (6–31). However, this study also did not show developmental improvements from ages 13 to 25, even in the TD group. Visual inspection of the graph suggests that performance may start to improve typically around age 25; at this point, the group with ASD may begin to diverge from the TD group (see Figure 1 in The EU-AIMS Leap Group et al., 2020a).

Similar to face recognition in those with ASD (discussed on p. 3, and perhaps also face perception; Stantiæ et al., 2022), recognition of emotional facial expressions becomes more impaired from adolescence to adulthood in those with ASD, as their TD peers improve on these tasks. A meta-analysis reported that individuals with ASD, compared to TD individuals, were impaired at recognizing all types of emotions, although some emotional expressions were more impacted than others (Lozier et al., 2014). Much like our conclusions on face recognition, Lozier and colleagues (Lozier et al., 2014) conclude: “The results of this meta-analysis provided strong evidence that individuals with ASDs are significantly impaired in recognizing multiple emotional facial expressions and that these deficits increase in magnitude over the course of development (p. 8, 9).” Their results indicate a lack of adolescent development (“essentially flat”) in those with ASD. These authors also suggest that age explains the inconsistent effect sizes in the literature on atypical expression recognition in ASD. In addition to the lack of development in the recognition of other people’s expressions, another meta-analysis indicates that deficits in emotional self-awareness in individuals with ASD emerge around adolescence. Emotional self-awareness, generally assessed by self-reported alexithymia measures, did not differ between children with and without ASD before the age of 12. However, individuals with ASD become less aware of their own emotions from adolescence into adulthood, while TD adolescents and adults do not exhibit age-related changes (see Figure 6 in Huggins et al., 2021). This decrease in emotional self-awareness is likely to undermine emotion regulation, an important skill, by adulthood in those with ASD (Reyes et al., 2019).

The pattern of arrested development in adolescents with ASD is, as in our studies, not evident across all tasks. A meta-analysis examining sensitivity to biological motion indicated that this social cognitive ability is impaired in individuals with ASD; however, it also appears to improve *more with age* in those with ASD, relative to their TD peers (Todorova et al., 2019). Another meta-analysis indicated that there were group differences between ASD and TD on motion processing, but these deficits were stable across age and were not specific to biological motion (and therefore not to social cognition). These differences instead reflected decreased sensitivity to motion in general (Van der Hallen et al., 2019).

### Neuroimaging studies

Studies have long indicated that young children with ASD display atypical developmental trajectories of brain maturation, compared to TD children (Courchesne et al., 2001, 2003, 2007). Aypical developmental mechanisms, distinct learning processes, and unique experiences are all likely to alter the maturation of brain function and structure in those with ASD. Even if the endpoint is similar, the pattern of maturation has important behavioral implications. Atypical trajectories of brain function and structure during childhood and adolescence in ASD are associated with clinical outcome in adulthood (Murphy et al., 2011), and with autism symptomology (The EU-AIMS Leap Group et al., 2020b).

#### Brain function

##### Activation during EF, decision making, and cognitive control tasks

Several neuroimaging studies have assessed whether brain function during EF and related tasks (with non-social stimuli) differs between groups with ASD and TD groups. A meta-analysis analyzed activation underlying three components of EF, namely inhibition, updating, and switching, across multiple neuroimaging studies. These authors used an activation likelihood estimation (ALE) meta-analysis to examine how these EF components overlap in individuals with ASD compared to their peers with TD (7–57; Zhang et al., 2020). The foci of activation underlying inhibition did not differ between children and adolescents, with or without ASD. However, the foci *did* differ between adults with ASD (who relied more on R inferior frontal gyrus) and TD adults (who relied more on L medial frontal gyrus). There were no reported group or age differences in the analyses of regions underlying updating or switching.

Two cross-sectional studies examined whether activation important for decision making differed between a group with ASD and a matched TD group. These studies found that the activation underlying both sustained attention (Murphy et al., 2014) and temporal discounting (i.e., inhibition + planning; Murphy et al., 2017) increased with age typically (Males, 11–35). However, in both studies, the group with ASD did not exhibit the increasing activation with age that was evident in the TD group. The increasing activation with age in the TD group, but not in the group with ASD, was evident in inferior and middle frontal cortices and striato-thalamic regions during the sustained attention task (Murphy et al., 2014) and ventromedial frontal cortex and cerebellum during the temporal discounting task (Murphy et al., 2017). There were also overall group differences, with lower activation across the group with ASD—compared to the TD group—during sustained attention in prefrontal cortex (PFC), striato-thalamic, and lateral cerebellar regions; and during the delay in temporal discounting in R ventrolateral/dorsolateral PFC, ventromedial PFC, striatolimbic regions, and cerebellum. On both tasks, the atypical maturation of activation and the overall differences in activation in these regions were associated with performance on the task, and with clinical measures of symptoms related to ASD.

##### Activation during social cognitive tasks

Much of the neuroimaging work in ASD on social cognition has focused on activation during face recognition and its well-known neural substrates (Haxby et al., 2000), in particular the fusiform face area (FFA) in ventral temporal lobe (Kanwisher et al., 1997). FFA appears to be less active when viewing individual faces in adults with ASD compared to TD adults, under some circumstances (Schultz et al., 2000, 2003; Koshino et al., 2008; Patriquin et al., 2016). However, FFA activation in ASD may be relatively typical with familiar faces (Pierce et al., 2004). The decreases in FFA activation in ASD may also be mediated in part by atypical gaze patterns often described in ASD during face perception or recognition (Hadjikhani et al., 2004, 2007; Dalton et al., 2005). Less is known about whether group differences between adults with and without ASD also reflects that the maturation of FFA that occurs typically, with activation increasing from childhood to adulthood (Scherf et al., 2007; Golarai et al., 2007, 2010; O’Hearn et al., 2011b), does not proceed typically in ASD. Studies suggest that the maturation of FFA activation and connectivity that occurs typically from adolescence to adulthood may be disrupted in individuals with ASD (Lynn et al., 2018; O’Hearn et al., 2020; see also Scherf et al., 2010).

A representational similarity analysis (RSA; Kriegeskorte et al., 2008), much like a multi-voxel pattern analyses (MVPA), was used to examine activation during face and car recognition on the CFMT. We assessed whether the patterns of activation (the voxels above threshold during memorization only) for distinct but within-category exemplars overlapped more with age. The categories included faces (American or novel Norwegian faces, analyzed separately; McKone et al., 2012) and cars (O’Hearn et al., 2020). Activation patterns for within-category exemplars did become more similar, with RSA scores increasing from adolescence to adulthood, in the TD group. The increased RSA score occurred in both the R (functionally-defined) FFA, and in structurally defined regions of interest in the L inferior frontal gyrus, bilateral temporoparietal junction, L inferior temporal lobule, and R fusiform gyrus. In contrast, the group with ASD displayed no changes in the RSA score in the FFA or any of these structurally defined regions from adolescence to adulthood. This change in the TD group but not in the group with ASD resulted in group differences by adulthood. In addition, RSA within-category score in the FFA, collected when memorizing exemplars, was positively correlated with subsequent recognition performance during test trials in both groups. This result is consistent with previous results in memory studies, which indicated increased overlap in the multi-voxel pattern analysis (MVPA) was associated with better memory performance in TD adults (Xue et al., 2010).

As part of the Longitudinal European Autism Project (LEAP), individuals with ASD and TD peers watched shapes move in either animated or non-animated manners. While there were robust effects of task type (animate vs. inanimate), there were no effects of age or diagnosis on functional activation in regions underlying social reasoning, similar to their behavioral results (ages 6–3) (see section “Behavioral studies of social cognition and communication,” p. 5, The EU-AIMS Leap Group et al., 2020a).

##### Connectivity during social cognitive tasks

A number of studies have examined the functional connectivity underlying face recognition in groups with ASD compared to TD groups, across both children and adults (Just et al., 2012; Supekar et al., 2013). These early studies suggested that connectivity was atypical in those with ASD. A connectivity study from our group examined task-based functional connectivity between the functionally-defined FFA region of interest and the rest of the brain during face and car recognition (Lynn et al., 2018). During face recognition only, analyses of FFA connectivity resulted in a significant interaction between group and age, in connectivity from R FFA to R dorsal striatum/temporoparietal junction, L dorsal anterior cingulate cortex, and thalamus—and from L FFA to R dorsal striatum. This interaction resulted from underconnectivity in children with ASD becoming overconnectivity in adults with ASD, compared to their TD peers. During face *and* car recognition, analyses revealed a similar pattern of atypical age-related changes in FFA connectivity to the amygdala in ASD, with underconnectivity in children and overconnectivity in adults compared to the TD group. Mamashli and colleagues also used a face (and house) processing paradigm. They have previously reported reduced FFA connectivity to striatal and superior temporal regions in adolescents and young adults with ASD (14–21), compared to their peers with TD (Khan et al., 2013). More recently, they did the same task with younger children with and without ASD (7–13). In this study, there were no group differences between the TD group and the group with ASD in FFA connectivity. This suggests that FFA connectivity becomes more atypical with age in ASD (Mamashli et al., 2018).

##### Connectivity during rest

Resting state functional connectivity has become a popular method for examining connectivity without the confounds (or the advantages) of task-based studies, such as different levels of performance and distinct baselines between groups. Our group explored resting-state functional connectivity from structurally defined striatal ROIs to the whole brain (Padmanabhan et al., 2013). These analyses indicated that connectivity increased with age in those with ASD and decreased with age in their TD peers. This pattern occured in the connections between the striatum (caudate, putamen) and posterior temporal regions (e.g., fusiform gyrus, inferior and superior temporal gyri; also from ventral striatum to anterior aspects of cerebellum). Children with ASD displayed decreased connectivity compared to TD children, but this difference was reversed by adulthood, leading to significantly increased connectivity in adults with ASD compared to TD adults.

Other papers have examined already established networks that develop into adulthood, and are known to differ in developmental disorders. The networks examined typically are often those important for EF and cognitive control (Fair et al., 2009, 2012; Dosenbach et al., 2010; Grayson and Fair, 2017). To examine how age-related changes in functional connectivity during rest differ between TD groups and those with ASD, a selective review analyzed resting-state functional connectivity in cross-sectional studies. This review reported that connectivity within and between the salience, default mode, and central executive networks develops differently in ASD compared to TD groups, potentially affecting cognitive control in those with ASD (Solomon et al., 2017). A longitudinal study indicated that, while TD individuals displayed increased segregation between these three networks over about three years (initial age 11–14), individuals with ASD did not exhibit increased segregation between these networks with age during this time frame, reflecting a lack of maturation in young adolescents with ASD (Lawrence et al., 2019). Finally, another longitudinal study reported important correlates of functional connectivity measures in the salience network, default-mode network, and frontoparietal control network (i.e., central executive). Connectivity of these networks predicted changes in autistic traits and adaptive behavior approximately 2 years later in those with ASD (Plitt et al., 2015).

#### Brain structure

##### Gray matter thickness

Gray matter thickness currently provides a particularly robust measure of adolescent development in brain structure. Thus, this measure may be particularly sensitive to differences between TD adolescents and adolescents with ASD (Wallace et al., 2015; Tamnes et al., 2017). Changes in gray matter thickness in temporal, frontal and parietal regions peak during adolescence (Gogtay et al., 2004; Gogtay and Thompson, 2010). For these reasons, other papers have argued that it is misleading to collapse across a range of ages when examining group differences (ASD vs. TD) in gray matter thickness (Greimel et al., 2013; Lin et al., 2015).

In TD individuals, the pattern of gray matter maturation is altered by many factors including IQ, gender and stress (Sowell et al., 2004; Shaw et al., 2006, 2020). In those with ASD, the pattern of maturation is also altered by IQ and gender, but these alterations *differ* from the changes in TD groups (Misaki et al., 2012; Wallace et al., 2013; Mrc Aims Consortium et al., 2020; Yankowitz et al., 2020). Examining developmental differences is further complicated because the pattern of gray matter maturation appears to be more idiosyncratic in those with ASD than in TD peers (Zabihi et al., 2019), potentially reflecting subtypes of ASD (Rommelse et al., 2017; van Rooij et al., 2018; The EU-AIMS Leap Group et al., 2020c).

Group differences in the pattern of gray matter thinning between individuals with ASD and their TD peers vary across regions, and are most notable in the frontal and temporal regions that undergo a prolonged typical trajectory (Boedhoe et al., 2020; Lukito et al., 2020). These group differences can also change direction with age, with thicker cortex in children with ASD becoming thinner cortex in adults with ASD, relative to each group of TD peers (Prigge et al., 2021; Yankowitz et al., 2021; but see Riddle et al., 2017). Such results are consistent with a cross-sectional study that revealed accelerated age-related cortical thinning in L temporal regions in those with ASD, compared to matched TD sample (12–24; Wallace et al., 2010). This work was followed by a longitudinal study indicating that cortical thinning was accelerated in both temporal and parietal cortices during adolescence in ASD compared to TD peers (14–24; Wallace et al., 2015). In contrast, The EU-AIMS Leap Group et al. (2020b) found no group differences in voxel-based analyses of gray matter thickness between groups with ASD and TD groups. However, this analysis, did not directly examine age and covaried or regressed out age (6–30), IQ, and a number of other factors. In contrast, ICA analysis resulted in interesting overall group differences in this study.

Trajectories of gray matter thinning from the large ENIGMA dataset indicate that the group differences between individuals with ASD and their TD peers (2–64) are greatest during adolescence. However, these changing group differences continue into adulthood. By adulthood, the superior frontal cortex is thicker and the temporal pole is thinner in those with ASD compared to TD adults (van Rooij et al., 2018). Young adults with ASD (18–24) also exhibited accelerated gray matter thinning, primarily in L temporal lobes, compared to TD adults. In this study, gray matter was thicker in the frontal regions underlying EF and higher-order cognition, and thinner in temporal and nearby parietal regions (supramarginal gyrus) related to social cognition and interaction in young adults with ASD compared to TD peers (Braden and Riecken, 2019). This result is consistent with those from a cross-disorder meta-analysis, again from the Enigma group. This analysis reported that, in adulthood, frontal cortices are thicker in groups with ASD compared not only to TD group but also to groups with attention deficit hyperactivity disorder (ADHD) or obsessive-compulsive disorder (OCD) (Boedhoe et al., 2020).

##### White matter integrity

Diffusion Weighted Imaging (DWI) examines the “integrity” of white matter tracts by assessing how water diffuses along the axons that form the tracts. Increased integrity is measured by increased fractional anisotropy (FA; water diffusion along the white matter tract) and decreased radial diffusivity (RD; water diffusion orthogonal to the white matter tract), providing a measure of efficiency and a proxy assessment of myelination. White matter integrity appears to increase linearly throughout childhood and adolescence, peaking during adulthood in a tract specific manner, and then declining later in life in TD individuals (Westlye et al., 2010). The white matter tracts that mature late include the uncinate fasciculus (temporal to orbitofrontal), the superior longitudinal fasciculus (temporal to inferior frontal), and the corpus callosum (across hemispheres; Asato et al., 2010).

The trajectory of white matter maturation is unclear in those with ASD. Overall, white matter integrity is most often reported to be reduced in samples with ASD compared to TD peers (Solomon et al., 2017). Not surprisingly, differences in ASD are most evident in late maturing tracts (e.g., uncinate fasciculus and superior longitudinal fasciculus, which connect the temporal and frontal cortices). However, collapsing across age in analyses of white matter integrity may distort the results, as group differences again appear to change with age (Karahanoğlu et al., 2018). While white matter integrity may increase in a linear fashion into adulthood in TD individuals, one study indicates the maturational trajectory of white matter into adulthood is essentially flat in those with ASD (Karahanoğlu et al., 2018). However, this result contradicts results from Thompson and colleagues, who found that white matter integrity increases with age in both those with ASD and their TD peers, and this relationship was significantly *stronger* in those with ASD. They report that white matter maturation is delayed in children with ASD, but then it catches up, becoming more similar to TD children during adolescence (Thompson et al., 2020).

## Discussion

Differences between groups with and without ASD can vary based on the age of the participants. Thus, focusing on developmental change and brain maturation is often critical for accurately characterizing samples with ASD. This review describes how developmental changes are often arrested or atypical during adolescence and early adulthood in individuals with ASD, compared to the continued developmental improvement often evident in their TD peers. This pattern was reported on measures of daily living skills, EF, social cognition and communication, emotional recognition, and emotional self-awareness. Skill in these domains is critical during the adolescent transition, and atypical adolescent development is likely to undermine adult outcomes in ASD.

An atypical trajectory in adolescents with ASD compared to TD peers is also apparent in neuroimaging studies of brain activation, connectivity, and structure. Atypical brain maturation in those with ASD has been reported in functional activation and connectivity during decision-making, EF, and social cognitive tasks; functional connectivity during rest; and brain structure, in particular gray matter thickness and white matter integrity. Differences in these neuroimaging measures between groups with and without ASD were most often evident in the temporal, frontal, and striatal regions, and the connections between these regions. The pattern of gray matter maturation in those with ASD was distinct from the pattern in TD individuals well into adulthood, and were also unique compared to samples with other developmental disorders (Boedhoe et al., 2020).

A better understanding of how and why differences that characterize ASD change with age will clarify the developmental mechanisms altered in ASD. In addition, information on the association between distinct behavioral trajectories and atypical brain maturation in those with ASD, and how those associations differ in TD individuals, will provide insight into whether individuals with ASD are compensating for their differences in particular ways (e.g., using more prefrontal cortex with age to process social scenes) as well as how behavioral development and brain maturation interact over time and development. If not maturing typically, do the differences in behavioral skills and associated brain maturation become progressively more atypical into adulthood in those with ASD?

This paper highlights the need to thoroughly understand the interconnected trajectories of behavioral development and brain maturation, including the “TD” trajectory, before making conclusions about differences in developmental disorders. The TD trajectory for most tasks is not well-delineated. It may be longest for important social tasks, such as face and emotion recognition (Germine et al., 2011; Lozier et al., 2014). This prolonged typical development of social skills is consistent with evidence that brain structure in temporal and frontal lobes is the last to mature (Gogtay et al., 2004; Asato et al., 2010). TD individuals may become able to rapidly interpret social information during adolescence, as they become increasingly reliant on global (vs. local) visual processing to interpret complex scenes (Kovacs, 2000; Shore et al., 2006; Scherf et al., 2009). Because of this increased reliance on global processing with age, differences may emerge during adolescence between TD individuals and peers with ASD, who are often characterized as focusing on processing the “local” features within a scene (Dakin and Frith, 2005; Mottron et al., 2006). Thus, visuospatial abilities in ASD may be relatively typical early in childhood, compared to TD individuals, prior to the maturation of global processing strategies that support improved performance in adulthood typically (Scherf et al., 2008; O’Hearn et al., 2013). Individuals with ASD may exhibit a lack of development in social skills during adolescence for multiple related reasons, including a lack of global processing and/or attention to social stimuli (Wagner et al., 2018), as well as a lack of the relevant experiences and/or disrupted acquisition of social expertise during adolescence.

ASD is likely to change how development itself works. Developmentalists have long examined how innate skills interact with experience, and how this interaction leads to the emergence of further capabilities. Greenough et al. (1987) did early work on experience-expectant and experience-dependent visual plasticity. Both of these developmental mechanisms are likely to be impacted by ASD. Experience-expectant mechanisms are adapted for input that is experienced by almost every individual, and thus may have a more “hard-wired” neural architecture that is sensitive within a specific developmental window (Andersen, 2003; McLaughlin and Gabard-Durnam, 2022). An example of this type of plasticity is evident in children born with congenital cataracts that later have them removed. These children acquire many visual abilities when the cataracts are removed but still struggle with the fine-grain discrimination needed for face recognition (Maurer et al., 2007). It has been suggested that children with ASD may display similar difficulties, due to a lack of attention to faces early in development (Chevallier et al., 2012; Wagner et al., 2018). In contrast, experience-dependent mechanisms allows mammals to adjust to their individual experiences over time, potentially through the generation of novel patterns of synaptic connections. This type of plasticity may lead to differences in the characteristics of ASD across cultures. Much like age, cultural differences are also likely to contribute to the contradictory results in the literature on visual differences in ASD. Koh and Milne (2012) report cross-cultural differences between individuals with ASD in the UK and those in Singapore. The often-reported focus on features or individual elements in ASD (i.e., weak central coherence or field-independence) was evident in the sample from the UK but not in the sample from Singapore.

More work is needed to identify if the atypical adolescent development in ASD reflects “sleeper effects” or experience-expectant development that is not getting the appropriate input at the correct time (Maurer et al., 2007); systematically different adolescent experiences in those with ASD or experience-dependent development (Orsmond et al., 2013); the interaction of these two types of development (Nelson, 2001); or some other developmental mechanism. Despite these continuing questions focusing on age and development will provide much needed insight into the inconsistent literature on ASD and, more importantly, the mechanisms that result in atypical development in individuals with ASD into adulthood. This paper underscores the need to directly examine age and/or developmental stage in almost all studies of ASD. We may need to understand additional factors like culture or country and etiology before making generalizations about differences in behavior or brain function/structure in ASD. This is a daunting task and highlights the need for attempted replication of results across samples, which is clearly critical to the study of ASD but underutilized. Further developmental research will provide insight into the developmental mechanisms that differ in ASD, and which mechanisms contribute to the poor outcomes evident in many adults with ASD.

## Author contributions

KO’H wrote this manuscript and led the studies attributed to “our laboratory” in the text. AL helped with the writing and all other aspects of the manuscript. Both authors contributed to the article and approved the submitted version.
